# Enhanced detonators detection in X-ray baggage inspection by image manipulation and deep convolutional neural networks

**DOI:** 10.1038/s41598-023-41651-y

**Published:** 2023-08-31

**Authors:** Lynda Oulhissane, Mostefa Merah, Simona Moldovanu, Luminita Moraru

**Affiliations:** 1 Laboratory of Signals and Systems (LSS), Faculty of Science and Technology, Abdelhamid Ibn Badis University of Mostaganem, 11 Route Nationale, Kharouba, 27000 Mostaganem, Algeria; 2 Department of Computer Science and Information Technology, Faculty of Automation, Computers, Electrical Engineering and Electronics, Dunărea de Jos University of Galati, 2 Stiintei Str., 800146 Galati, Romania; 3 Modelling & Simulation Laboratory MSlab, Dunărea de Jos University of Galati, 47, 800008 Galati, Romania; 4 Department of Chemistry, Physics and Environment, Faculty of Sciences and Environment, Dunărea de Jos University of Galati, 47 Domneasca Str., 800008 Galati, Romania

**Keywords:** Engineering, Aerospace engineering, Electrical and electronic engineering

## Abstract

Detecting detonators is a challenging task because they can be easily mis-classified as being a harmless organic mass, especially in high baggage throughput scenarios. Of particular interest is the focus on automated security X-ray analysis for detonators detection. The complex security scenarios require increasingly advanced combinations of computer-assisted vision. We propose an extensive set of experiments to evaluate the ability of Convolutional Neural Network (CNN) models to detect detonators, when the quality of the input images has been altered through manipulation. We leverage recent advances in the field of wavelet transforms and established CNN architectures—as both of these can be used for object detection. Various methods of image manipulation are used and further, the performance of detection is evaluated. Both raw X-ray images and manipulated images with the Contrast Limited Adaptive Histogram Equalization (CLAHE), wavelet transform-based methods and the mixed CLAHE RGB-wavelet method were analyzed. The results showed that a significant number of operations, such as: edges enhancements, altered color information or different frequency components provided by wavelet transforms, can be used to differentiate between almost similar features. It was found that the wavelet-based CNN achieved the higher detection performance. Overall, this performance illustrates the potential for a combined use of the manipulation methods and deep CNNs for airport security applications.

## Introduction

The detection of dangerous objects in X-ray images of baggage has become important, particularly due to rising crime rates^1^. The performance of screening devices is strongly influenced by the target visibility, image display technology, and the security officers’ knowledge. However, visual inspection of these images is highly challenging due to the low prevalence of targets, variability in target visibility (resulting in lack of precision in object shape), overlapping objects, poor contrast that obscures image details, and the potential for causing false alarms^2,3^. Furthermore, the constant and repetitive nature of the task i.e., the security officers constantly looking at screens and frequently encountering the same types of detected objects, can lead to attention fatigue and impaired judgment^4^.

The most dangerous prohibited articles in passenger baggage are so called improvised explosive devices. Detecting the detonator of a bomb can be a challenge even for well-trained security officers. To address these issues, numerous algorithms and techniques have been developed to improve the quality of 2D radiographic images^5–13^. The Bag-of-Visual-Words (BoVW) detection technique, which is based on natural language processing and information retrieval, employs a statistical process for object detection and classification^6^. This technique has been successfully applied for the detection of explosives. It was used together with various other methods, including supervised feature learning by autoencoders approach^7^, K-Nearest Neighbors, Logistic Regression^8^, and Decision Trees^9^. BoVW was also employed for the detection of guns, shuriken or razor blades. These techniques are based on dictionaries formed for each class and the detection consists of Scale Invariant Feature Transform (SIFT) feature descriptors of randomly cropped image patches^10^. The BoVW model correlated to the Speeded up Robust Features (SURF) descriptor and Support Vector Machine (SVM) classifier was used for firearm detection, achieving an optimal true positive rate of 99.07% at a false positive rate of 20%^11^. Both random forest and SVM algorithms were used for firearm detection and a statistical accuracy of 94% was reported^12^. Single, two and multiple X-Ray views and four classifiers (i.e., Scale-Invariant Feature Transform, Oriented FAST and Rotated BRIEF, Binary Robust Invariant Scalable Keypoints and SURF) were considered to assess the performance of classification. A better performance of classification has been highlighted when a combination of two and multiple X-Ray views was considered^13^.

In recent years, CNNs have gained significant popularity in the field of X-ray image analysis for baggage screening^14–20^. The augmentation technique, a feature enhancement module and a multi-scale fused region of interest method enabled the development of new CNNs with more accurate and robust detection capabilities. Those CNNs have a significantly improved performance when dealing with densely cluttered backgrounds during the X-ray baggage inspection^14^. Various techniques were employed to overcome different shortcomings of deep CNNs which were caused by a shortage of training images. Thus, the transfer learning paradigm^15,16^, region-based CNN (R-CNN), mask-based CNN (Mask R-CNN) and detection architectures such as RetinaNet were used to provide object localization variants^17,18^ or to detect various items in the X-ray image of the baggage. In a similar fashion, the You Only Look Once (YOLO) architecture was used for X-ray images of baggage classification and for hazardous materials identification^19^. Moreover, an anchor-free CNN-based object detection method was proposed to address the problem of dangerous objects detection^20^.

Wavelet transforms are a popular tool in image denoising. They are mostly used in denoising operations without any prior knowledge of the noise model. Also, they are a useful tool for image enhancement. Apart from image denoising, which is a subjective process, image enhancement alters the image features to make it more appealing to the human eye^21^. Wavelet edges effects are noticeable in the processed images but wavelets are not currently used in X-ray security inspection. Implementation of the wavelet transforms for X-ray security inspection is still lacking even if they are extensively used in several contexts of chest X-ray images, due to their strong predictive capabilities. Various studies reported only the implementation of machine learning or deep learning radiomics models for predicting COVID-19 prognosis, based on edge detection or radiomic features extraction^22–24^. While the utilization of wavelet transforms with CNNs in medical image processing has been extensively studied, their application in other domains, such as aviation and transport security, has been relatively limited.

In the case of X-ray airport security data, large amounts of training data are not always available and collecting X-ray data with the special request of data annotation is very expensive. A study devoted to the performance of screeners’ assessment in detecting bomb detonators with 2D and 3D imaging was conducted in Ref.^25^. Despite the lower image quality in 3D imaging, this study found that the performance was almost similar to that of 2D imaging. In another approach, an Unsharp masking USM + CLAHE algorithm to process radiographic images for airport security was developed to effectively overcome the color distortion induced by the CLAHE image enhancement^26^. Generally, the majority of the previous studies have primarily focused on the detection of secondary explosive devices (such as C4, TNT, etc.) in X-ray images during baggage inspection. Nowadays, there are limited studies that specifically address the detection of detonators in the X-ray baggage inspection process. For instance, a Dual-Energy method was used to detect dangerous objects, including detonators, by differentiating between organic and inorganic materials^27^. The majority of existing scientific publications dealing with this topic focus on various algorithms devoted to enhancing the image quality and to improve the detection performance of detonators^28^, but few concentrate on wavelet-based detonators detection in X-ray images.

The aim of this paper is to introduce a new and efficient scheme for detecting detonators in X-ray baggage images by comparing different image manipulation methods and by evaluating their impact on the predictive capabilities of the classification models. To overcome the underutilization of wavelets in X-ray security inspections, we introduce wavelets as a manipulation method, which can be used to obtain images with higher resolution and more defined details. This allowed us to gain insights into the validity of the manipulation processes and how they relate to the performance of detonators detection. The experiments are conducted using the High Tech Detection Systems (HTDS) database. In the proposed approach we have chosen and built a well-established CNN architecture which had achieved excellent performance in object classification and detection^29^. We have conducted an extensive ablation study to establish an optimal configuration model with good performance across the dataset^30,31^. Thus, we have experimented with different images corrupted by Gaussian and salt-and-pepper noise, various altered hyper-parameters and different layer structures. The proposed CNN architectures perform two stages of analysis: (i) detonators detection within the raw X-ray image using the deep CNN-based classifier, the TensorFlow and Keras libraries and, (ii) the same CNN classifier framework is used when the input image set is pre-processed using the following methods: the CLAHE algorithm, which operates independently on the RGB images and furthermore, also on the individual color channels, the wavelet transforms with the HH and HL sub-bands, and, a combination of CLAHE and RGB-wavelet transform techniques. The outputs are analyzed in terms of accuracy, precision, recall, F1-score, and classification.

Our novel contributions compared to other state-of-the-art approaches can be summarized as follows:

- We proposed a multiscale approach by combining CLAHE, wavelet transforms, and RGB-wavelet transforms with CNNs to address the issue that different X-ray image quality factors can make the detection of the detonators a difficult task.
- We conduct experiments on manipulated images to find the proper technique able to achieve the highest detection performance.
- The custom CNN architecture proved compatible with various image manipulation techniques, being able to exploit the distinguishability between classes of baggage, with and without detonators inside.

The proposed methods of image manipulation emulate various technical specifications and assess the detection performance. Besides its practical relevance, a comparison of these manipulation methods is also of theoretical interest. The proposed study validates wavelets as a new framework for further studies in multivariate multiresolution analysis of X-ray screening of passenger baggage.

In our opinion, these experiments are at the proof-of concept level. We have tried to demonstrate that our idea could be turned into a reality. However, at this stage, only relatively limited datasets are available for sound training and testing operation.

## Results

In this paper, we have investigated the ways in which the manipulation of the quality of X-ray images can create a feature map which leads to an improved discrimination of dangerous materials, like detonators. The deep CNN architecture works with the TensorFlow and Keras libraries, based on the High Tech Detection Systems (HTDS) database. The deep CNN only has two classes, namely detonators and non-detonators. We used the augmentation techniques to increase the number of samples and to avoid overfitting. The final dataset consists of 15,115 samples of which 4535 samples were used for our tests. A random 70/30 training/testing splits of the dataset has been performed. Due to sample scarcity in the original dataset, we used fivefold cross-validation during the training of raw X-ray images. The same CNN architecture was set up for all experiments.

Table 1 presents the binary classification performance for the raw image dataset. A classification accuracy of 0.9808 is reported. All detonator spot predictions are correct.

**Table 1:** Detection results of the deep CNN for raw X-ray images.

	Accuracy	Classification	Precision	Recall	F1-score	Confusion matrix
X-ray image original	0.9808	0	1.00	0.97	0.99	$$\left[\begin{array}{cc}1344& 37\\ 0& 549\end{array}\right]$$
		1	0.94	1.00	0.97	

**Figure 1:**
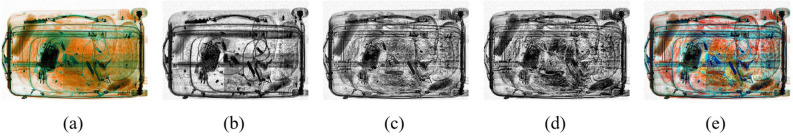
CLAHE enhancement of the original radiographic image and its results for each color channel for an RGB image (**a**) original; (**b**) Red channel; (**c**) Green channel; (**d**) Blue channel; (**e**) RGB image.

To analyze the effects of the contrast enhancement and wavelet transformation on the detection efficiency, the performance of the classification for each proposed approach was examined. A visual representation of how the radiographic image has been improved by using the CLAHE method is shown in Fig. 1. The results of the CLAHE image enhancement algorithm are presented in Table 2.

**Table 2:** Detection results produced by the deep CNN when the CLAHE image enhancement method and color channel decomposition are employed.

CLAHE	Accuracy	Precision	Recall	F1-score	Confusion matrix
Red channel	0.996	1.00	0.99	0.99	$$\left[\begin{array}{cc}3766& 17\\ 0& 550\end{array}\right]$$
Green channel	1.00	1.00	1.00	1.00	$$\left[\begin{array}{cc}3783& 0\\ 0& 550\end{array}\right]$$
Blue channel	1.00	1.00	1.00	1.00	$$\left[\begin{array}{cc}3783& 0\\ 0& 550\end{array}\right]$$
RGB image	1.00	1.00	1.00	1.00	$$\left[\begin{array}{cc}3783& 0\\ 0& 551\end{array}\right]$$

The CLAHE method is working very well on the green and blue channels, as well as on RGB images. In both cases, it has achieved an accuracy of 1.00. These values are higher than the accuracy of the raw radiographic images (Table 1), with a difference of 1.92%. All detonator spot predictions are correct for the G and B channels and for the RGB images. While it may be unrealistic to expect 100% accuracy values, we note that the CLAHE can add the discrimination potential by improving the contrast and image quality and by reducing the loss of details in the image. However, the number of TP samples (images with detonator) is lower.

**Figure 2:**
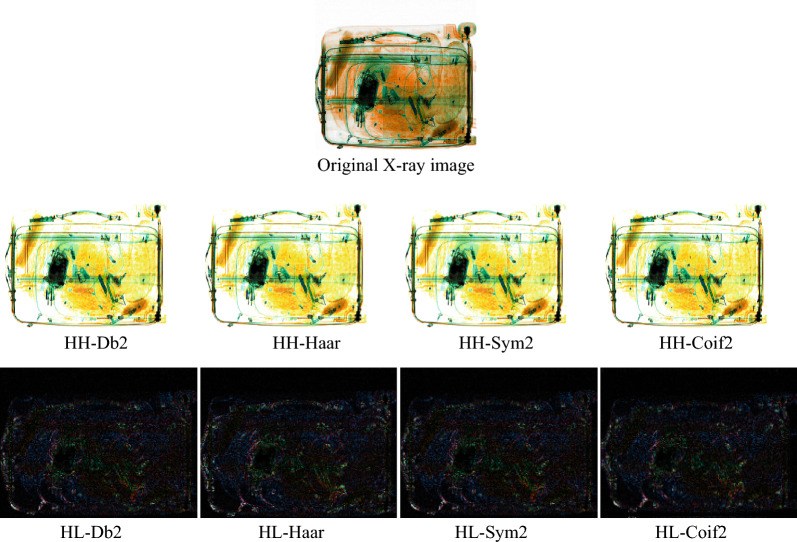
Wavelet transformation of the original radiographic image and the obtained results for two sub-bands, HH and HL. Daubechies2 (Db2), Haar, Coiflet2 (Coif2), and Symlet2 (Sym2) wavelet functions are used for the transformation.

To examine the effects of decomposing an image into a set of wavelets and to analyze the improvements of the local spectral and temporal information extraction, the X-ray image decomposition using the Haar, Db2, Coif2, and Sym2 wavelet functions has been performed. The HL and HH details coefficients or sub-bands are of interest. The results are shown in Fig. 2 and details regarding the performance of classification are presented in Table 3.

**Table 3:** Detection results of the deep CNN for wavelet transforms applied for the HH and HL sub-bands.

Level	Wavelet transforms	Accuracy	Precision	Recall	F1-score	Confusion matrix
HH	Haar	0.986	1.00	0.98	0.99	$$\left[\begin{array}{cc}3922& 63\\ 0& 550\end{array}\right]$$
	Sym2	0.994	1.00	0.99	0.99	$$\left[\begin{array}{cc}3347& 21\\ 1& 549\end{array}\right]$$
	Db2	0.990	0.99	0.98	0.99	$$\left[\begin{array}{cc}3824& 41\\ 1& 549\end{array}\right]$$
	Coif2	0.995	0.99	0.99	0.99	$$\left[\begin{array}{cc}3824& 1\\ 19& 531\end{array}\right]$$
HL	Haar	1.00	1.00	1.00	1.00	$$\left[\begin{array}{cc}3952& 0\\ 0& 549\end{array}\right]$$
	Sym2	0.995	0.99	1.00	0.99	$$\left[\begin{array}{cc}3931& 21\\ 0& 549\end{array}\right]$$
	Db2	0.999	0.99	1.00	0.99	$$\left[\begin{array}{cc}3951& 1\\ 0& 549\end{array}\right]$$
	Coif2	0.998	0.99	0.99	0.99	$$\left[\begin{array}{cc}4020& 7\\ 2& 498\end{array}\right]$$

As it can be seen, the Coif2 function at the level HH achieved an accuracy of 0.995, which is slightly higher than Sym2 (0.994) and higher than Db2 and Haar (0.990 and 0.986). At the HL level, the Haar function resulted in an accuracy of 1.00, surpassing all the other functions: Db2 (0.999), Coif2 (0.998), and Sym2 (0.995).

Additionally, the data in Table 3 shows that the accuracy results provided by wavelet transforms are superior to the accuracy obtained when raw radiographic images are directly fed to the CNN (Table 1). It also shows that the Haar transform has the same performance as the color channel decomposition in terms of accuracy (100%). However, the number of true positive TP samples (images with detonators) is smaller in the case of the RGB channel decomposition, while the number of true negative TN sample is similar.

**Figure 3:**
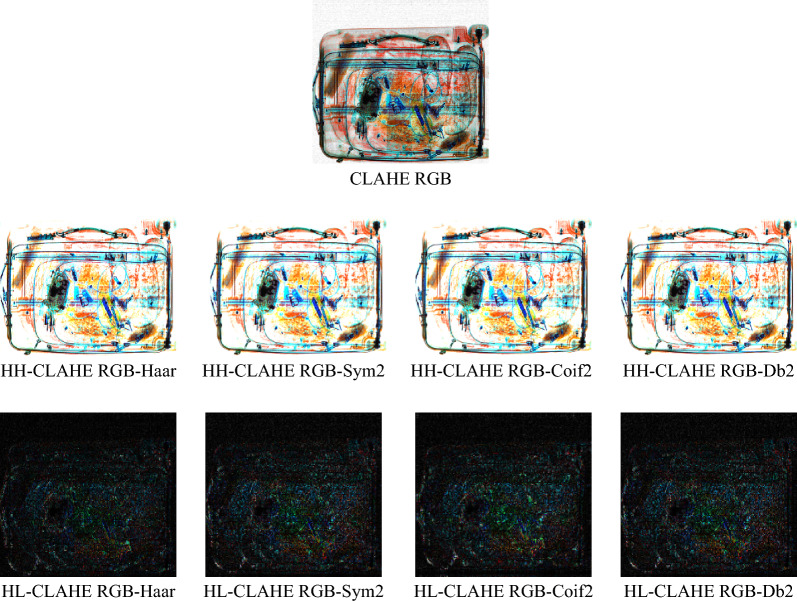
Image preprocessing using the CLAHE RGB and wavelet transforms. The X-ray image in the RGB space is contrast enhanced using CLAHE. The result is decomposed through the instrumentality of the wavelet transform applied in the HH and HL sub-bands.

When both the CLAHE RGB and wavelet transformation methods are combined, the result of pre-processing is shown in Fig. 3. A summary of the performance of classification is given in Table 4.

**Table 4:** The performance of classification for the CLAHE RGB-Wavelet methods at the HH and HL sub-bands.

Level	CLAHE RGB-wavelet	Accuracy	Precision	Recall	F1-score	Confusion matrix
HH	Haar	0.999	0.99	0.99	0.99	$$\left[\begin{array}{cc}3645& 1\\ 1& 548\end{array}\right]$$
	Sym2	0.992	0.99	1.00	0.99	$$\left[\begin{array}{cc}3612& 33\\ 0& 549\end{array}\right]$$
	Coif2	0.997	0.99	1.00	0.99	$$\left[\begin{array}{cc}3633& 13\\ 0& 549\end{array}\right]$$
	Db2	0.999	0.99	0.99	0.99	$$\left[\begin{array}{cc}3640& 6\\ 1& 548\end{array}\right]$$
HL	Haar	0.999	1.00	0.99	0.99	$$\left[\begin{array}{cc}3651& 0\\ 3& 547\end{array}\right]$$
	Coif2	0.999	0.99	0.99	0.99	$$\left[\begin{array}{cc}3644& 7\\ 2& 498\end{array}\right]$$
	Sym2	0.995	0.99	0.99	0.99	$$\left[\begin{array}{cc}3640& 11\\ 6& 494\end{array}\right]$$
	Db2	0.996	0.99	0.99	0.99	$$\left[\begin{array}{cc}3651& 0\\ 4& 550\end{array}\right]$$

It can be observed that the Haar wavelet at the HH level achieved an accuracy of 0.999. This is the highest accuracy among the other mother wavelet functions, namely Db2 (0.999), Coif2 (0.997), and Sym2 (0.992). For the HL level, the Haar and Coif2 wavelets obtained an accuracy of 0.999, which is still the highest when compared to the Db2 (0.996) and Sym2 (0.995).

**Figure 4:**
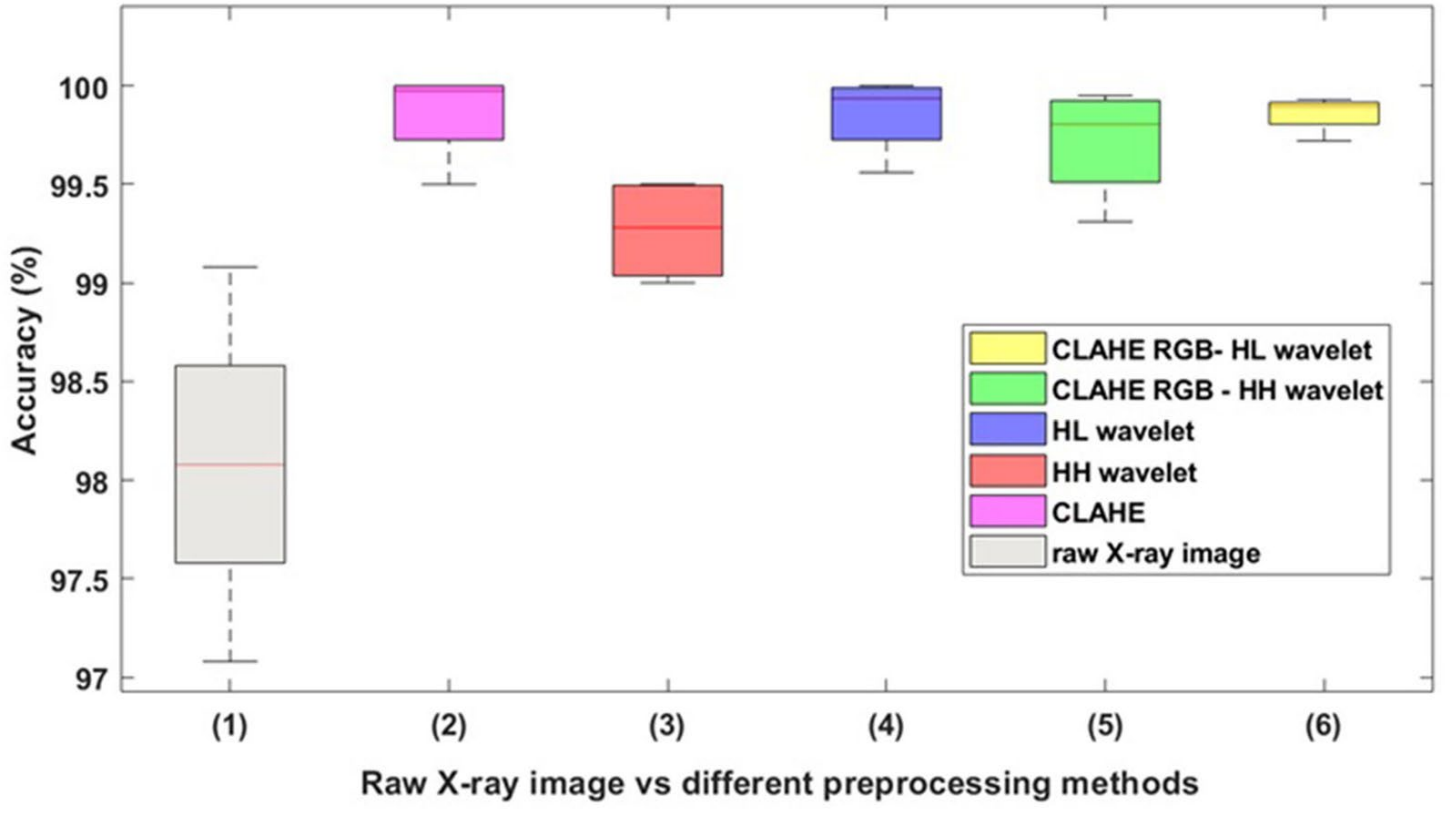
CNN average accuracy of classification for raw X-ray images, and as well as the accuracies of the proposed preprocessing models. The central lines indicate the median accuracy values and the boxes indicate the interquartile range. The whiskers indicate the smallest and largest values. Both the raw X-ray images and HL wavelet processed images have zero skew for the accuracy distribution. The rest of the methods show accuracies with a slightly negatively skewed distribution. Overall, we obtained the best performance with the Haar wavelet transform at HL sub-band along with the CLAHE & RGB image method.

Generally, the accuracy of classification obtained using the CLAHE RGB-wavelet method is significantly higher that the accuracy obtained for raw X-ray images, but almost similar to the results provided by the wavelet decomposition. We emphasize once again, that although the performance metrics have very good values, they alone are not enough to indicate any enhancement in the detection of detonators. In the wavelet decomposition case, the number of TP samples (images with detonators) is higher than in the case of RGB channel decomposition or wavelet transform while the number of true negative (TN samples) is almost identical. There is an insignificant number of FN samples (i.e., type II error) in the classification. To summarize the findings in our study, a descriptive statistic of the accuracy is provided in in Fig. 4.

## Discussion

Although many researchers have used classical methods for contrast enhancement, we introduce wavelets as a manipulation method to obtain images with higher resolution and more defined details. With this approach, we investigated the accuracy of detection when there are subtle differences in the image details, i.e., edges enhancements, altered color information or different frequency components provided by wavelet transforms. All of these could be used to differentiate between almost similar features in images. We have achieved improved classification results by exploiting high-level features provided by wavelet transforms. The first key result presented in this paper is that the presence of detonators can be effectively discriminated by traditional image processing methods such as wavelet decomposition or by combining contrast enhancement techniques and Haar wavelet transforms at the HL sub-band combined with deep CNN model. The use of the deep CNNs for detonators detection resulted in an improved recall (the ability to identify the positive class aka images with detonators) and precision (how accurate the positive predictions are).

Although deep CNNs have been a booster for images classification, we are aware that both the quality of an image, together with the max-pooling down-sampling method may dilute or remove some features that are important for classification. In order to determine the optimal architecture and configuration of our CNN model, an ablation study has been performed for a clear understanding of the model’s performance. The consequence of altering some components or hyper-parameters was a decrease in the performance of the model. The current network architecture provides an optimal performance with low computational complexity (see Tables 6 and 7). The proposed CNN uses a built-in mechanism to extract different high-level features and introduce spatial invariance. Also, the networks learning abilities are improved by its structure. The convolution layers result in different image features being extracted to a different extent and the pooling operation builds the ability to detect the same feature in different images. By feeding the same CNN architecture with different variants of the original image (i.e., various features are highlighted by manipulation) the net can be more optimally trained and its pattern recognition ability and image interpretation performance are thus improved.

The wavelet transforms ensure an effective Gaussian noise removal and they can extract the detail of images pretty well. The HH and HL components contain textural representations of the original image. It is worth noting that, when we used different frequency components and different features of the colour channels, more informative features were extracted and a better performance of detection was achieved. We were able to capture fine and coarse details of the image and improve image quality. The CLAHE method performs local histogram equalization pixel by pixel, thus improving both the contrast and image quality. On the other hand, the wavelet transform method generally recovers features from regions of interest and processes the image in pairs of pixels.

For dual X-ray images, the color information could be relevant to the detection. We have noticed that RGB images shows better results. Certain colors are indicative of the presence or absence of prohibited items and the model can learn these relationships. It's important to note that in some cases, converting RGB images to grayscale or to color channels can introduce biases and distortions. In this case, the enhancement method could fail to balance the areas with uneven illumination and some details became invisible as subtle color deviations appear.

It can be seen that, unlike the original images, images processed with the CLAHE RGB-Wavelet method (HH and HL sub-bands) and wavelet transforms at HH and HL sub-bands suffer from false negatives and from false positives. Images processed with the CLAHE RGB and Haar wavelet (HL sub-band) suffer from neither false positives nor false negatives. Our preliminary results show that the CLAHE & RGB image methods (Table 2), along with the Haar wavelet transform applied for the HL sub-band (Table 3) achieve a 100% true positive rate. This means that these methods are well-suited for detonators identification through the CNN approach. The differences (albeit very small) between the image manipulation results in detonators detection are explained as follows: the contrast enhancement works using the intensity map of pixels, reduces the loss of details and doesn’t modify the pixel position in the image. CLAHE applies color balancing through histogram adjustment. The contribution of the local spectral and temporal information to the edge extraction provided by the wavelet transforms is considered. Usually, the noise information exists in the high-frequency component and the low-frequency component contains the relevant information about the image. HL images balance both aspects and CLAHE better reduces the noise in the HL sub-band.

To summarize the findings in our study, we have observed that increasing the number of preprocessing tasks (we refer here to the CLAHE—RGB—Haar wavelet and the HH and HL sub-bands) does not necessarily lead to an increase in accuracy. These high-frequency HH and HL sub-bands saved most of the information so that the CNN could preserve more features and provide an accurate classification.

Table 5 reports the comparison of the accuracy values reported in our work and other classification approaches and the detection strategies based on deep CNN architectures, CLAHE enhancement and wavelet transforms. We compare our experimental results with some previous studies that experimented with the same methods but with different images type and databases (only accuracies). For fair comparisons, we conducted the experiments on detonator detection using two pre-trained models, EfficientNetV2B0 and AlexNet. Both are pre-trained using the ImageNet database, which contains millions of labeled images.

**Table 5:** Performance comparison of the proposed work compared to other works on CNN architecture from the literature.

References	Image modality	Method	Accuracy
^32^	X-ray images	Wavelet based Deep CNN: the DWT featured sub-images are input to the CNN algorithm	0.9887
^33^	X-ray images	CNN the VGG16 learning architecture: CLAHE + RGB + Gaussian filter and thresholding images	0.9875
^34^	X-ray images	Wavelet transforms + contrastive learning + COVID-Net model	0.9355
^35^	X-ray images	CLAHE, CNN	0.9100
Proposed work	X-ray images	CLAHE (Green channel + Blue channel + RGB image), Deep CNN	1.00
		Wavelet (HH-Haar-CLAHE RGB), Deep CNN	0.9995
		Wavelet (HH-Wavelet Coif2), Deep CNN	0.9950
		EfficientNetV2B0	0.9920
		AlexNet	0.9945

As shown in Table 5, although the AlexNet has a high accuracy (0.9945), it generates a number of false positives (16 on average). The EfficientNetV2B0 has the worst performance, while our network outperforms it regardless of how the images are manipulated. These results indicate the robustness of our approach.

Our experiments are at the proof-of-concept level, but they demonstrate that our idea could be turned into reality. However, at this stage, there are some limitations. The dataset was relatively small to train our CNN. Data augmentation was our method of choice to deal with overfitting, but we are also devoted to the entropic capacity of our CNN model, that is, the amount of information that the CNN model can store. The architecture of our CNN model allows storing a large amount of information, so its potential to be more accurate by leveraging more features was increased. Another limitation could rise from the cluttered nature of the X-ray image input dataset. This could cause the CNN to fail to detect the detonators. The proposed solution demonstrates that the CNN is an accurate tool as the FN samples occurrence is insignificant (Tables 3, 4). A more complex and complete study (more images, various sizes for baggage, features extraction specific to the organic sample, another neural network type etc.) will be carried out in the future. Also, it would be interesting to extend the research to multiclass classification, including other dangerous objects in X-ray images such as, TNT, C4, and PBX (plastic-bonded explosives).

## Methodology

**Figure 5:**
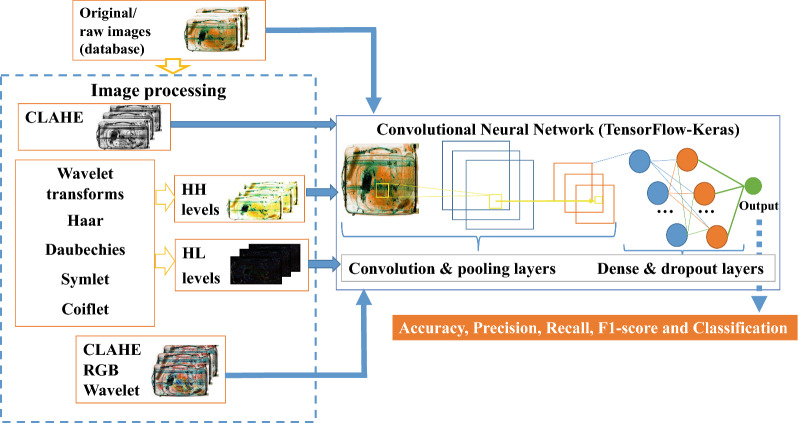
Block diagram of the proposed method after the ablation study. HH (High-High) and HL (High-Low) are two sub-bands of decomposition in wavelet transform.

In order to detect suspicious objects, such as detonators, in the 2D radiographic images of baggage from the HTDS database, the proposed block diagram in Fig. 5 outlines the experiments conducted.

### Database

**Figure 6:**
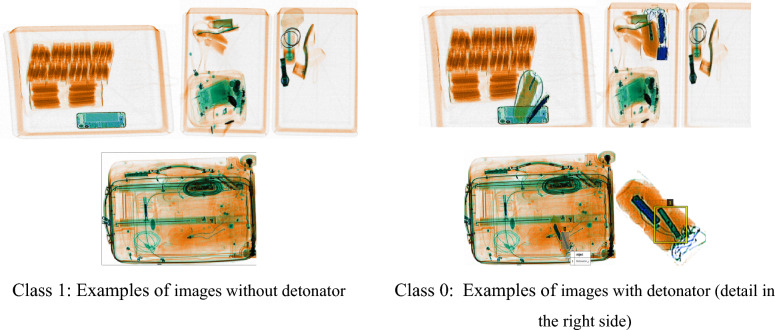
Examples of X-ray images of baggage with multiple objects (top row) and without/with detonator inside (bottom row). The detail of a detonator is illustrated. Different materials shown in different colors on a pseudo-color image from dual-energy X-ray scans illustrate the 2D image low quality and resolution.

The database used in this study is sourced from High Tech Detection Systems (HTDS), a French company specialized in the sales and maintenance of high-tech security equipment used for passenger screening, baggage and vehicle security, and freight^36^. The initial dataset consists of 6,500 images divided into two types: class 0, which consists of 5,500 images with detonators and class 1, which consists of 1,000 images without detonators, as shown in Fig. 6.

The dataset has been split into training (70%) and test sets (30%) such that each split has a similar class distribution. The test set contains samples never used for training. To balance the number of samples in both classes we have performed random rotations to each sample to augment the dataset. The final dataset consists of 15,115 samples. The performance of classification is assessed on the test dataset.

### Image manipulation

The CLAHE enhancement method is used to improve the image quality and contrast in the dual-energy radiographic images^23^. The contrast can be manipulated by modifying the intensity map of pixels. It optimizes the object identification, preserves details through morphological processing, and performs RGB (red, green, blue) conversion.

Discrete wavelet transforms can extract spectral features. A 2D wavelet transform passes the image through a low-pass and high-pass filter and approximate and detailed parts are provided. This multi-resolution framework generates four sub-bands, namely one approximation coefficient (LL) and three detail coefficients, horizontal (HL), vertical (LH), and diagonal (HH), respectively. The LL sub-band contains no edge and is not useful for our goal. Both the HL and LH sub-bands contain almost similar information and for the sake of efficiency, only the HL is investigated. To this end, we used the following mother functions: Haar, Daubechies2 (Db2), Symlet2 (Sym2), and Coiflet2 (Coif2)^21,22^. During the wavelet transforms, the input image is convolved with low-pass and high-pass filters and down sampled to obtain the wavelet sub-bands. Only the first level wavelet decomposition is used. Both the HL and HH images are passed through hidden convolution layers, then batch normalization allows faster execution and solves the issue of poor convergence. Finally, a softmax layer is used to categorize the dual-energy radiographic images. To assess the capability of the pre-processed images to be meaningful for the deep CNN classification task, we also combine the CLAHE—RGB conversion with HH and HL sub-band wavelet transformations, using the same mother functions.

### Ablation study

The ablation study allows a clear understanding of the proposed model by analyzing the consequences of altering images and components of the CNN architecture. For fair comparisons, the raw and wavelet transformed images from the HTDS database were used. The results of the entire ablation study are recorded in the Tables 6 and 7. To evaluate the performance of the ablation process, the same metrics (accuracy, precision, recall and F1-score) were used.

**Table 6:** Ablation study on the raw images database.

Ablation elements		Accuracy	Precision	Recall	F1-score	Confusion matrix
Input images	Image corrupted by Gaussian noise	0.8746	0.91	0.90	0.91	$$\left[\begin{array}{cc}1256& 110\\ 132& 432\end{array}\right]$$
	Image corrupted by salt and paper noise	0.8958	0.93	0.91	0.92	$$\left[\begin{array}{cc}1285& 92\\ 109& 444\end{array}\right]$$
Number of convolution layers	1	0.8708	0.93	0.91	0.92	$$\left[\begin{array}{cc}3304& 237\\ 305& 350\end{array}\right]$$
	2	0.9159	0.96	0.94	0.95	$$\left[\begin{array}{cc}3404& 138\\ 215& 441\end{array}\right]$$
Learning rate	0.01	0.9256	0.95	0.95	0.96	$$\left[\begin{array}{cc}3421& 149\\ 163& 463\end{array}\right]$$
	0.005	0.9338	0.95	0.97	0.96	$$\left[\begin{array}{cc}3451& 164\\ 102& 478\end{array}\right]$$
Batch size	16	0.9423	0.94	0.97	0.96	$$\left[\begin{array}{cc}3499& 163\\ 81& 502\end{array}\right]$$
	64	0.9427	0.96	0.96	0.96	$$\left[\begin{array}{cc}3466& 121\\ 119& 489\end{array}\right]$$

**Table 7:** Ablation study on HH and HL images.

Metrics	Number of convolution layers			Learning rate				Batch size			Number of convolution layers			Learning rate				Batch size	
	1	2		0.01		0.005		16		64	1	2		0.01		0.005		16	64
Haar wavelet HH level											Haar wavelet HL level								
Accuracy	0.945	0.945	0.922		0.926		0.926		0.902		0.933	0.931	0.926		0.919		0.902		0.917
Precision	0.967	0.964	0.937		0.941		0.959		0.958		0.967	0.968	0.950		0.955		0.953		0.957
Recall	0.969	0.972	0.971		0.971		0.951		0.922		0.955	0.950	0.964		0.946		0.929		0.941
F1-score	0.968	0.968	0.954		0.956		0.955		0.939		0.960	0.959	0.957		0.951		0.941		0.949
Sym2 wavelet HH level											Sym2 wavelet HL level								
Accuracy	0.937	0.957	0.930		0.919		0.919		0.917		0.940	0.943	0.946		0.943		0.949		0.941
Precision	0.961	0.965	0.944		0.944		0.955		0.943		0.972	0.968	0.969		0.957		0.953		0.960
Recall	0.965	0.986	0.974		0.974		0.946		0.959		0.957	0.965	0.968		0.976		0.987		0.971
F1-score	0.963	0.975	0.959		0.959		0.951		0.951		0.965	0.966	0.951		0.67		0.970		0.965
Coif2 wavelet HH level											Coif2 wavelet HH level								
Accuracy	0.961	0.968	0.954		0.941		0.936		0.925		0.953	0.943	0.943		0.967		0.951		0.959
Precision	0.962	0.978	0.972		0.958		0.946		0.942		0.970	0.968	0.968		0.985		0.960		0.977
Recall	0.993	0.986	0.975		0.973		0.978		0.971		0.975	0.965	0.965		0.976		0.983		0.975
F1-score	0.977	0.981	0.974		0.966		0.961		0.956		0.972	0.966	0.967		0.980		0.971		0.976
Db2 wavelet HH level											Db2 wavelet HH level								
Accuracy	0.957	0.960	0.952		0.967		0.950		0.954		0.960	0.947	0.956		0.965		0.951		0.956
Precision	0.965	0.975	0.966		0.986		0.966		0.961		0.966	0.972	0.981		0.948		0.959		0.976
Recall	0.985	0.980	0.977		0.977		0.975		0.985		0.987	0.965	0.969		0.974		0.983		0.972
F1-score	0.975	0.977	0.972		0.981		0.971		0.973		0.976	0.968	0.974		0.979		0.971		0.974

It can be seen that the CNN + wavelet transform models, for all wavelet families, achieves better accuracy values and outperforms the CNN model which used raw images as input.

### CNN classification

We performed extensive experiments to examine the strength of the proposed CNN model for the binary classification of dual-energy radiographic images into two classes: detonator and non-detonator. The CNN architecture is implemented using TensorFlow and Keras. TensorFlow is a free and open-source software library for data flow and differentiable programming through a variety of functions. It is very convenient and flexible for building the current deep learning models^37^. Keras is an open-source neural network library written in Python and runs on top of TensorFlow^38^. The deep CNN is entirely based on five deeply separable layers (i.e., three convolutional layers and two dense layers). Table 8 shows the proposed hyperparameters for the deep CNN architecture model, after the ablation study. The first convolutional layer has 16 units, the second layer has 32 units, and the third layer has 64 units. The Rectified Linear Unit (ReLU) activation function is used, as it is a non-linear function and has the advantage of avoiding backpropagation errors. The Adam (Adaptive Moment Estimation) optimizer is used with the default learning rate of 0.001 and 1e − 6 decay. The batch size of 32 is the optimal solution. The right number of epochs depends on the inherent complexity of the dataset. We had employed six values for the epoch (60, 80, 100, 120, 140, 160) in an attempt to improve our model. The number of epochs without improvement in loss function plateau was between 55 and 65, for all experiments. The time per epoch is between 72 and 76 s, with an average loss of 0.0677. We have determined that a good time to stop training is around epoch 60. The accuracy of the proposed CNN with 60 epochs has been chosen as the optimal option with less time and reduced losses. Dense layers are fully connected layers that transform one 1D feature vector into a classification vector. They are followed by the ReLU activation functions. The last dense layer does not have a specified activation function because the logit values are used and the classification is performed using a softmax activation function.

**Table 8:** Hyperparameters of the deep CNN architecture.

Layer (type)	Output shape (No of filters)
Input	(None,200,200,3)
conv2d_1(Conv2D)	(None,200,200,3)
max_pooling2D_1(MaxPooling2D)	(None,200,200,16)
conv2d_2(Conv2D)	(None,100,100,16)
max_pooling2d_1 (MaxPooling2D)	(None,100,100,32)
conv2d_3 (Conv2D)	(None,50,50,32)
max_pooling2d_2 (MaxPooling2D)	(None,50,50,64)
dense_1 (Dense) + ReLU	(None,85)
dropout_1 (Dropout)	(None,50,50,64)
dense_2 (Dense)	(None,2)
Batch size	32
Learning rate	0.001
Number of epochs	60

For baselines comparisons, we quantify our proposed CNNs in terms of effectiveness and efficiency with two commonly used models, EfficientNetV2B0 and AlexNet. All the networks are trained using the same datasets for fair comparisons.

### Evaluation

The performance of the classification is evaluated comparing the average accuracy, precision, recall and F1-score, using same test dataset and proposed manipulation methods. These measures were calculated using the confusion matrix binary classification problems (i.e., a 2 × 2 matrix). The binary confusion matrix provides accurate insights into the model's performance and determines if the model can effectively discriminate between classes and identify specific prediction errors, which can lead to model improvements or adjustments. The confusion matrix includes the following elements: TP = true positives, TN = true negatives, FP = false positives, FN = false negatives. The performance metrics derived from this matric are: recall = [TP/(TP + FN)], precision = [TP/(TP + FP)], accuracy = [(TP + TN)/(TP + TN + FP + FN)]), F1-score = 2[(precision × recall)/(precision + recall)]. All results are averaged over the test dataset. The average accuracy of the detonator detection over all samples indicates how many of our detonator and non-detonator predictions are correct. The average recall indicates how many of the detonator samples are predicted as such and the average precision shows how many of our detonator predictions are correct.

## Data Availability

The dataset is available upon request from High Tech Detection Systems (HTDS, https://www.htds.fr/). The data are not publicly available. The data that support the findings of this study are available from the first author, [LO], upon reasonable request.

## Code availability

Source code is made available at https://gitfront.io/r/user-3214156/oGn8DHKwkwzB/inspection-by-image/.
